# Sustained silencing peanut allergy by xanthopurpurin is associated with suppression of peripheral and bone marrow IgE-producing B cell

**DOI:** 10.3389/fimmu.2024.1299484

**Published:** 2024-02-06

**Authors:** Nan Yang, Kamal Srivastava, Yujuan Chen, Hang Li, Anish Maskey, Patrick Yoo, Xiaohong Liu, Raj K. Tiwari, Jan Geliebter, Anna Nowak-Wegrzyn, Jixun Zhan, Xiu-Min Li

**Affiliations:** ^1^ R & D Division, General Nutraceutical Technology, LLC, Elmsford, NY, United States; ^2^ School of Life Science and Technology, Changchun University of Science and Technology, Changchun, Jilin, China; ^3^ Central Lab, Shenzhen Bao’an Chinese Medicine Hospital, Shenzhen, China; ^4^ Department of Pathology, Microbiology and Immunology, New York Medical College, Valhalla, NY, United States; ^5^ Department of Pediatrics, Icahn School of Medicine at Mount Sinai, New York, NY, United States; ^6^ Department of Respiratory, The First Affiliated Hospital of Guangzhou University of Chinese Medicine, Guangzhou, China; ^7^ Department of Pediatrics, Hassenfeld Children’s Hospital, NYU Grossman School of Medicine, New York, NY, United States; ^8^ Department of Biological Engineering, Utah State University, Logan, UT, United States

**Keywords:** *Rubia cordifolia* L., food allergy, IgE, transcriptome, RNA-Seq

## Abstract

**Introduction:**

Peanut allergy is an immunoglobulin E (IgE) mediated food allergy. *Rubia cordifolia* L. (*R. cordifolia*), a Chinese herbal medicine, protects against peanut-induced anaphylaxis by suppressing IgE production *in vivo*. This study aims to identify IgE-inhibitory compounds from the water extract of *R. cordifolia* and investigate the underlying mechanisms using *in vitro* and *in vivo* models.

**Methods:**

Compounds were isolated from *R. cordifolia* water extract and their bioactivity on IgE production was assessed using a human myeloma U266 cell line. The purified active compound, xanthopurpurin (XPP), was identified by LC-MS and NMR. Peanut-allergic C3H/HeJ mice were orally administered with or without XPP at 200µg or 400µg per mouse per day for 4 weeks. Serum peanut-specific IgE levels, symptom scores, body temperatures, and plasma histamine levels were measured at challenge. Cytokines in splenocyte cultures were determined by ELISA, and IgE ^+^ B cells were analyzed by flow cytometry. Acute and sub-chronic toxicity were evaluated. IL-4 promoter DNA methylation, RNA-Seq, and qPCR analysis were performed to determine the regulatory mechanisms of XPP.

**Results:**

XPP significantly and dose-dependently suppressed the IgE production in U266 cells. XPP significantly reduced peanut-specific IgE (>80%, p <0.01), and plasma histamine levels and protected the mice against peanut-allergic reactions in both early and late treatment experiments (p < 0.05, n=9). XPP showed a strong protective effect even 5 weeks after discontinuing the treatment. XPP significantly reduced the IL-4 level without affecting IgG or IgA and IFN-γ production. Flow cytometry data showed that XPP reduced peripheral and bone marrow IgE ^+^ B cells compared to the untreated group. XPP increased IL-4 promoter methylation. RNA-Seq and RT-PCR experiments revealed that XPP regulated the gene expression of CCND1, DUSP4, SDC1, ETS1, PTPRC, and IL6R, which are related to plasma cell IgE production. All safety testing results were in the normal range.

**Conclusions:**

XPP successfully protected peanut-allergic mice against peanut anaphylaxis by suppressing IgE production. XPP suppresses murine IgE-producing B cell numbers and inhibits IgE production and associated genes in human plasma cells. XPP may be a potential therapy for IgE-mediated food allergy.

## 1 Introduction

Food allergy (FA) is an adverse immune response to food allergens. It can be life-threatening and the prevalence of FA has significantly increased over the past decades, affecting about 32 million people in the United States, of which 5.6 million are children under age 18 (1–9). Treatment options are extremely limited. Food avoidance and rescue medication after accidental exposure are the first lines of FA management. Peanut allergy (PNA) causes severe, and sometimes fatal reactions, often co-existing with other food allergies (1, 10–15). Food allergy is also increasing in other westernized and developing countries (2, 16). It negatively influences the quality of life for patients and their families. Unfortunately, there are currently limited treatment options for food allergies. Strict avoidance of allergens remains the standard treatment for the majority of food allergies, except for peanut (PN). Since 2020, an FDA approved drug (Palforzia) is available for peanut desensitization in children 4-17 years old (17). However, it is not approved for other age groups due to lack of efficacy, and requires a prolonged course of treatment with indefinite daily dosing (18, 19). Palforzia is associated with a high rate of adverse effects and relapse after treatment cessation (20). Moreover, real-world acceptance of Palforzia is reported to be low (21). Therefore, the need to develop treatments, particularly for milk and peanut (PN) allergies, is urgent and challenging.

Food allergies typically are IgE-mediated. IgE molecules binding to mast cells and basophils sensitize these cells. When IgE specifically binds to the epitopes of the food allergen, it activates mast cells and basophils to release histamine and many other mediators (22). These, in turn, elicit inflammation and immediate hypersensitivity (22). Since IgE plays a pivotal role in food allergies, the concept of decreasing IgE levels has been investigated as a therapeutic strategy for food allergy treatment. In 2003, the anti-IgE antibody, TNX901, was tested in a double-blind, randomized trial. The results showed that TNX-901 significantly increased the threshold of the sensitivity of patients to peanut (23, 24). In another study, omalizumab (Xolair), was also found to greatly reduce anaphylactic reactions during the oral immunotherapy (OIT) (25). These studies suggested potential for developing novel therapies to regulate IgE. However, the current form of anti-IgE therapy has limitations. It demands continuing bi-weekly or monthly injections to maintain effects, as this treatment neutralizes secreted IgE, but does not stop B-cell IgE production (26, 27). In addition, biologic therapies are very expensive. An alternative and cost-effective approach to inhibiting IgE production by B cells is highly desirable.

Previously, Lopez et al. screened 70 herbal medicines from traditional Chinese medicine (TCM) and found that *Rubia cordifolia* L. (*R. cordifolia*) inhibited IgE production in a dose-dependent manner *in vitro* (28). *R. cordifolia* also suppressed the PN-specific IgE levels and protected PN-allergic mice against anaphylactic reactions (28). In this study, we aimed to test the effect of a pure compound, xanthopurpurin (XPP), isolated from *R. cordifolia*, in a PN-allergic murine model and investigate underlying mechanisms. We, for the first time, demonstrate that XPP markedly reduced peanut-specific IgE, protected peanut-allergic mice from anaphylaxis, and reduced plasma histamine levels. Splenocytes and bone marrow IgE-producing B cells were significantly reduced, which was associated with reduction of IL-4 and epigenetic modulation of IL-4 gene promoter. RNA-Seq analysis of plasma cells revealed that XXP has the capacity to regulate genes associated with antibody production.

## 2 Materials and methods

### 2.1 General materials

Water extract of *R. cordifolia* was purchased from E-Fong Herbs Inc. (South EI Monte, CA). Methanol, acetonitrile, and other chemicals were purchased from Fisher Scientifics (Pittsburgh, PA). Freshly ground, whole roasted PN (White Rose brand, NJ) were used to prepare the crude PN extract (CPE) as previously described (29, 30). Cholera toxin (CT) was purchased from List Biological Laboratories, Inc (Campbell, CA). The human myeloma cell line, U266 B cells, was purchased from American Type Culture Collection (Manassas, Virginia).

### 2.2 Isolation of active compounds from *Rubia cordifolia* L.

In order to isolate individual components from *R. cordifolia*, liquid-liquid extraction was used with different organic solvents. In brief, 200g of *R. cordifolia* water extract was first dissolved into 4L of DDH_2_O and extracted with 4L of dichloromethane for 24 hrs using a liquid-liquid extractor (Sigma-Aldrich, St. Louis, MO) at 100°C with a 2L heating mantle (EM2000/CX1, Barnstead International, Dubuque, IA). The dichloromethane extract of *R. cordifolia* was first dried using a rotary evaporator (Rotavapor R-210, BÜCHI, Switzerland), then redissolved into methanol and loaded onto Sephadex LH20 (Sigma-Aldrich, St. Louis, MO) column for further separation.

### 2.3 Liquid chromatography-mass spectrometer and NMR

Liquid chromatography was performed using a Waters 2690 HPLC-PDA system (Waters, Milford, MA). *R. cordifolia* water extract (30 mg/mL) was first filtered through a 0.8μm Nalgene™ syringe filter (Thermofisher, Waltham, MA) and separated on a ZORBAX SB-C18 (4.6 × 150mm, 5µm) column (Agilent, Santa Clara, CA) on the HPLC system. The separation was carried out using mobile phase A (0.1% aqueous formic acid) and mobile phase B (acetonitrile with 0.1% formic acid) at a flow rate of 1mL/min. The separation gradient started at 2% of mobile phase B to 25% within 45 min, to 35% within 25 min, to 55% mobile phase B within 15 min, to 75% in 10 min and maintained at 75% mobile phase B for another 5 min. Data was collected and processed using Waters’ Empower 2 software. Mass spectra data was collected using Waters Alliance 2695 HPLC system coupled with Waters LCT premier TOF mass spectrometer in both positive mode and negative mode to characterize the molecular weight of unknown constituent. The parameters were set as: Capillary Voltage: 3200 v; Cone Voltage: 15 v; Aperture I: 25 v; Desolvation Temperature: 300 ˚C; Source Temperature: 110 ˚C; Desolvation gas: 500 L/h; Nebulizing gas: 40 L/h; Ionization mode: Electrospray; Positive Ion Acquisition Range: m/z 50-1000. The results were collected and analyzed by Empower and Masslynx software. ^1^H NMR (at 300 MHz) and ^13^C NMR (at 75 MHz) spectra were obtained on a JOEL instrument using DMSO-*d_6_* as the solvent.

### 2.4 Human myeloma cell culture and IgE antibody measurement

The human myeloma cell line (U266), purchased from ATCC (American Type Culture Collection; Rockville, MD), has been used to test different herb extracts and purified compounds for their IgE inhibitory effects (31). 2×10^5^ cells/mL of cells were cultured at 37°C under 5% CO_2_ in complete media containing RPMI 1640 medium supplemented, 10% FBS, 1 mM sodium pyruvate, 1×10^-5^ M β-ME and 0.5% penicillin-streptomycin. XPP at 0, 2.5, 5, 10, 20 and 40 μg/mL were added at day 0. After 6 days, supernatants were harvested and IgE levels were measured by using an ELISA Kit (Mabtech Inc, OH). Briefly, samples and standards were added into prewashed 96-well plate coated with capture monoclonal antibodies (mAbs). After 2-hour incubation at room temperature, plate was washed 5 times and incubated with detection mAb for 1 hour. The plate was then washed for 5 times and incubated with streptavidin-HRP for 1hour at room temperature. The plate was then washed and developed with TMB substrate for 15 min. The optical density was measured using ELISA reader at 450nm. IgE concentrations were calculated based on the standard curve.

### 2.5 Animals

Six-week-old female C3H/HeJ mice were purchased from the Jackson Laboratory (Bar Harbor, ME). Mice were maintained on PN-free chow under specific pathogen-free conditions according to standard guidelines for the care and use of animals (32). The study protocol was approved by institutional animal care and use committee (IACUC) at Icahn Mount Sinai School of Medicine, New York (IACUC #LA11-00082). The institutional Public Health Service (PHS) animal welfare assurance number is D16-00069 (A3111-01).

### 2.6 Induction of PN allergic murine model and XPP treatment

**Figure 1:**
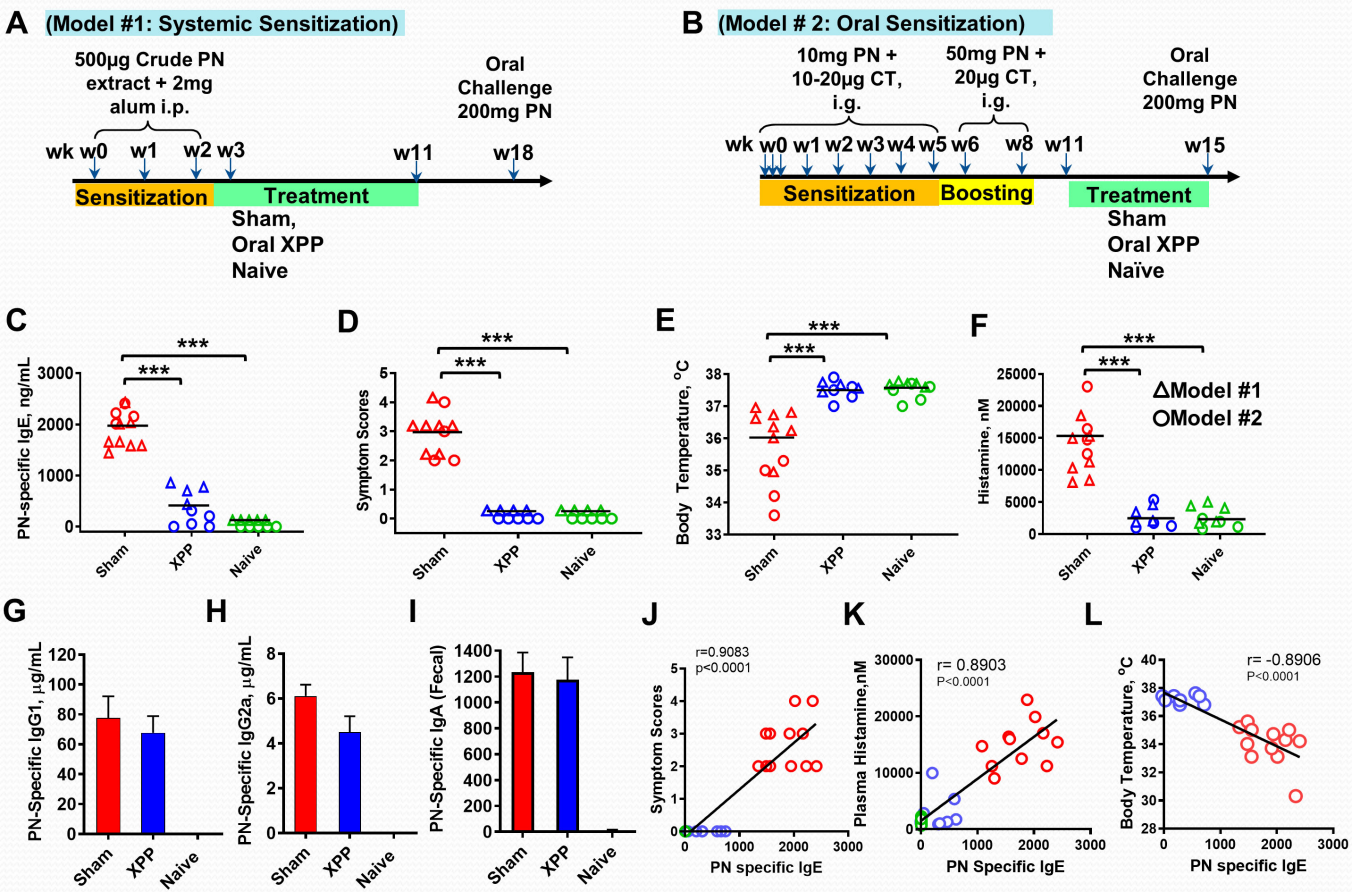
XPP prevented peanut-induced anaphylactic reactions in the systemic (triangle symbols) and Oral (circle symbols) sensitization protocols. **(A)** Experimental design for systemic i.p. PN sensitization; **(B)** Experimental design for oral PN sensitization; **(C)** Post-therapy PN-specific IgE in measure by ELISA; **(D)** Symptom Scores; **(E)** Body temperatures measured 30 minutes after PN challenge using a rectal probe; **(F)** Histamine levels in plasma obtained after PN challenge measured by ELISA; **(G)** Serum PN-specific IgG1 (Model #2); **(H)** Serum PN-specific IgG2a (Model #2); **(I)** Fecal PN-specific IgA (Model #2); **(J)** Correlation between PN specific IgE and Symptom score; **(K)** Correlation between Plasma histamine and PN specific IgE; **(L)**. Correlation between Body Temperature and PN specific IgE; Pearson correlation coefficient (r) was calculated. Bars indicate group means. *** = P < 0.001 *vs*. Sham. N = 4~5 mice/group from 2 separate experiments.

As described previously (33), mice were systemically sensitized by intraperitoneal (i.p.) injection of 500 μg of crude PN extract with 2 mg of alum in PBS (Model #1). Sensitization was given in three weekly injections at weeks 0, 1 and 2. XPP treatment started at week 3, representing an early treatment protocol as PN allergy is not yet established at this time point. Treatment with XPP (400 μg/day dissolved in drinking water) was given once daily for a period of 8 weeks. Sham mice were orally given water daily. Naïve mice were used as controls which were not sensitized or treated. Post therapy oral challenge was given at week 18 with 200 mg of PN (**Figure 1A**).

Late treatment with XPP was tested in an oral sensitization model (Model #2). As described previously (34, 35), C3H/HeJ mice were intragastrically (i.g.) sensitized three times at week 0 with 10 mg of homogenized PN extract in 0.5 mL PBS containing 75 mg sodium bicarbonate, 10 µg of the mucosal adjuvant cholera toxin (List Laboratories, CA), and 16.5 µL (1.1 µL/g body weight) of 80 proof of Stolichnaya Vodka^®^. After the first week sensitization, mice were given the sensitization solution weekly as above with 20 µg CT for 5 weeks. 50 mg PN with 20 µg CT was given as the boosting dose at weeks 6 and 8 using the same gavage solution. Naïve mice were not sensitized. Oral XPP treatment (400 μg/day dissolved in drinking water) started at week 11 and continued through week 15. This treatment protocol represents late treatment as PN allergy is established and mice are reactive to challenge at this timepoint as shown by us previously (34). Sham group of mice received water daily.

Oral challenge with 200 mg PN was given at the end of week 15. Mice were fasted for 2 hrs prior to sensitization and challenge (**Figure 1B**).

### 2.7 Assessment of hypersensitivity reactions

Anaphylactic symptoms were evaluated 30-40 minutes following oral PN challenge as described previously (34, 35). Visually observed symptoms were scored utilizing the scoring system described previously (34): 0 - no symptoms; 1 - scratching and rubbing around the snout and head; 2 - puffiness around the eyes and snout, pilar erection, reduced activity, and/or decreased activity with increased respiratory rate; 3 - wheezing, labored respiration, cyanosis around the mouth and the tail; 4 - no activity after prodding, or tremor and convulsion; 5 - death. Cage identities were concealed during visual assessment of anaphylactic symptoms. Rectal temperatures were measured using a rectal probe (Harvard Apparatus, NJ).

### 2.8 Measurement of PN specific-IgE, IgG1 and IgG2a

PN specific-IgE in serum was measured as reported previously (34–36). Briefly, 96-well microtiter plates were coated with crude PN extract (sample wells) and rat anti-mouse IgE (reference wells) and held overnight at 4°C. After washing three times, plates were blocked for 2 hours at room temperature with 2% BSA-PBS. After three washes plates were incubated with diluted serum samples and purified mouse IgE antibody (in reference wells) overnight at 4°C. Biotinylated anti-IgE was added to the plates followed with the addition of avidin-peroxidase and ABTS substrate. For PN specific-IgG1 or IgG2a ELISAs, plates were coated with DNP-HSA in the reference wells and CPE in the sample wells. After the blocking, anti DNP-IgG1 or anti DNP-IgG2a were added to the reference wells, while samples were added into the sample wells. Plates were developed with biotinylated Rat anti-mouse IgG1 or IgG2a (BD Biosciences, CA) and ABTS (LGC, Searcare, MA). All plates were read using a spectrophotometer (Molecular Devices, San Jose, CA) using SoftMax software (Molecular Devices, San Jose, CA).

### 2.9 Measurement of plasma histamine levels

As previously performed (34, 35), blood samples were collected via sub-mandibular bleeding at 30 minutes after scoring and measurement of body temperature and plasma was isolated immediately after the blood collection. All samples were stored at -80°C for further usage. Histamine levels in the serum were measured using a commercial available ELISA kit (Fisher Scientific, Waltham, MA) as described by the manufacturer.

### 2.10 Safety assays

For acute toxicity analysis, naïve C3H/HeJ mice were fed with 4 mg of XPP per mouse, which is10 times of the daily dose. Mice were observed for 14 days. In the sub-chronic toxicity assay, naïve C3H/HeJ mice were fed with 5 times their daily therapeutic dose (2 mg/day) for 14 days. Control group mice (sham) were fed with water. Blood samples were collected at the end of each experiment. Blood urea nitrogen (BUN) and alanine aminotransferase (ALT) measurements for the evaluation of kidney and liver functions respectively and complete blood count (CBC) testing were performed by ALX laboratories, NY.

### 2.11 Splenocytes cultures and cytokines measurement

Mice were sacrificed at the end of each experiment. The splenocytes (SPCs) and mesenteric lymph node (MLN) cells of each mouse was collected as previously described (37). Cells (4 x10^6^/well/mL) were cultured in 24 well plates in the presence or absence of CPE (200 µg/mL). After 72 hrs of incubation at 37°C under 5% CO_2_, supernatants were collected. Cytokine levels were measured using ELISA kits in triplicate according to the manufacturer’s instructions (BD Biosciences, San Jose, CA and R&D systems, Minneapolis, MN).

### 2.12 Cell viability

Cell viability was evaluated using a trypan blue exclusion as previously described (31). Briefly, a 10µL of cells suspension from each culture was mixed with equal volume of trypan blue dye. The mixture was loaded into a hemocytometer and cells were counted under a microscope. The percentage of viable cells was calculated as follows: Viable cells (%) = (total number of viable cells)/(total number of cells) × 100.

### 2.13 Flow cytometry

Single cell suspensions of spleen, bone marrow from two femurs and heparinized peripheral blood were isolated in AIM-V medium. Erythrocytes were lysed with red cell lysing buffer (Sigma-Aldrich, St. Louis, MO). For staining, cells were suspended in ice cold staining buffer (PBS including 0.5 mM EDTA, 0.05 mM Sodium Azide, 0.5% BSA). Surface staining was performed by incubating cells with unlabeled anti-IgE (to block membrane IgE), BV605 anti-B220, BV711-anti-CD3, anti-CD16/32 (Fc-block), all from BD Biosciences, CA). Live-dead discriminating dye (Live-Dead Aqua, Invitrogen, CA) was included. After incubation, cells were washed and suspended in fixation/permeabilization buffer for 15 mins. Cells were washed with permeabilization buffer and incubated with Fitc-anti IgE in permeabilization buffer for 30 mins in the dark on ice. After washing, cells were treated with Cytofix buffer for 15 mins for post-fixation and then washed 3 times with staining buffer and finally resuspended in 200 µL staining buffer for cell acquisition on LSRII flow cytometer (BD Bioscience, San Jose, CA). Flow cytometry analysis was performed using Flow Jo 5.4 software (Tree Star Inc., Ashland, Oregon) as follows. Singlet cells were selected on the basis of FSC-A/FSC-H profile and used to generate lymphocyte gate based on forward and side scatter properties (**Supplementary Figure 1**). Live cells were selected as cells negative for Live-Dead stain. From live cell gate, all IgE+ (Fitc-IgE) cells were gated and subsequently analyzed for IgE+ B cells (Fitc-IgE +; BV605-B220+ cells).

### 2.14 DNA methylation

Genomic DNA was isolated from mouse splenocytes collected from orally sensitized mouse model#2 using Qiagen mini DNA/RNA extraction kit (Qiagen, Valencia, CA). Isolated DNA was bisulfite-modified using an Epitect plus DNA Bisulfite kit (Qiagen) following the manufacturer’s instructions. Bisulfite converted DNA was then amplified with the IL-4 primers listed below by PCR. The PCR products were subsequently purified and pyrosequenced using sequencing primers on a Pyromark Q24 system (Qiagen) as described previously (38). Primers for IL-4; Fwd: GTTTTTAAGGGGTTTTTATAGTAGGAAG; Rev-Biotin-AATTACCACTAACTCTCCTCCTACA. Sequencing (CpG -393) AGATTTTTTTGATATTATTTTGTT.

### 2.15 RNA sequencing and identification of molecular targets underlying IgE regulation

U266 cells were treated with XPP at 10 μg/mL. After 3 days of treatment, cells were collected and total RNA was isolated using Qiagen RNeasy MiniKits following the manufacturer’s instructions. The purity of total RNA was analyzed and the RNA-Seq was performed with an Illumina HiSeq2500 instrument at the Genomics Core facility at Icahn School of Medicine at Mount Sinai. Counts were converted to Log2 counts per million and normalized. A log2 fold change was calculated and the gene list with values ≥ 2 or ≤ -2 were uploaded to DAVID bioinformatic system for functional analysis.

### 2.16 Real time polymerase chain reaction

RT-PCR analysis of genes related to Cell cycle, B cell differentiation, and IgE production was performed by QuantStudio 5 real-time PCR system (Thermo Fisher, Waltham, MA). For each PCR reaction, 12.5 μL maxima SYBR Green/ROX qPCR Master Mix 2x (Thermo Fisher, Waltham, MA) was mixed with 1.8 μL of 0.3 μM target primers and 300 ng of template DNA to make the total volume as 25 μL. The PCR procedure was set as 40 cycles at 25°C for 5 min, 42°C for 60 min and 70°C for 15 min. GAPDH was used as the housekeeping gene. The Delta Ct values for each gene were calculated by normalizing the Ct values with the housekeeping gene. The relative fold change in mRNA expression between different groups was calculated and expressed as 2^−ΔΔCT^. The primer sequence used are shown in **Supplementary Table 1**.

### 2.17 Statistics

Data were analyzed using GraphPad Prism software (version 8.2.1, GraphPad Software. La Jolla, CA). Differences between two groups were analyzed using unpaired Student’s t-test. Differences between multiple groups were analyzed by One Way Analysis of Variance (One way ANOVA) followed by pair wise testing using Bonferroni’s adjustment. Pearson’s correlation coefficients were analyzed using GraphPad Prism software, and r values were generated. P values ≤0.05 were considered statistically significant.

## 3 Results

### 3.1 XPP isolated from *R. cordifolia* dose-dependently inhibited IgE production by human IgE producing myeloma cell line

**Figure 2:**
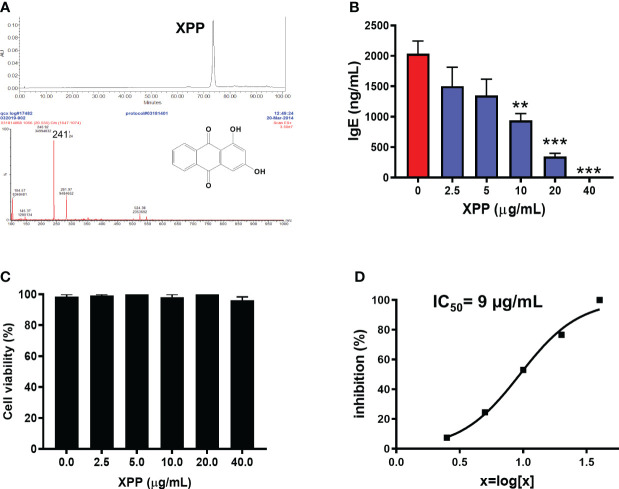
Characterization of xanthopurpurin (XPP) and the *in vitro* inhibitory effect of XPP on IgE by U266 cells. **(A)** HPLC chromatogram and Mass spectrum of XPP. The chemical structure of XPP was presented. **(B)** XPP dose-dependently inhibited IgE production by U266 cells (human IgE was measured by ELISA). **(C)** Cell viability assay of XPP on U266 cells by trypan blue exclusion. **(D)** IC50 values of XPP on IgE production in U266 cells. **p<0.01; ***p<0.001 vs. Untreated control (0 µg/mL). n=4-6.

*R. cordifolia* water extract was first extracted using dichloromethane, and further fractionated. Five fractions were collected and the fifth fraction (Fr.5) was found to have the highest anti-IgE effect at 10 µg/mL in human myeloma cell line (U266 cells) *in vitro* (**Supplementary Figure 2**). The major compound from this fraction was further collected and purified. The purity of the isolated compound was more than 95% as determined by analytical HPLC. The structure of this compound was identified using LC-MS (**Figure 2A**) and ^1^H and ^13^C NMR spectroscopy (**Supplementary Table 2**) (39). Mass spectra data showed a [M+H]^+^ ion peak of *m/z* 241 and [M-H]^-^ ion peak of *m/z* 239 (**Figure 2A**). The molecular weight (MW) of this compound was then determined to be 240 g/mol. The ^1^H NMR data and the ^13^C NMR data of this compound was consistent with previously reported data of xanthopurpurin (**Supplementary Table 2**). Thus, based on the MS and NMR data, this compound was identified as xanthopurpurin (XPP).

We determined the biological effect of XPP on IgE production *in vitro* on U266 cells at various concentrations. XPP inhibited the IgE production by U266 cells in a dose-dependent manner (**Figure 2B**). Inhibition of IgE was first observed at XPP concentration of at 2.5 µg/mL, reaching statistically significant inhibition at 10 µg/mL (p < 0.01), and completely inhibition of IgE production was observed at 40 µg/mL. Cell viability was analyzed using trypan blue staining as previously published (31). No toxicity was observed at any concentration tested (**Figure 2C**). The IC_50_ value was calculated as 9 µg/mL (**Figure 2D**). To our knowledge, this is the first report that XPP inhibits IgE production. Notably, there was no cytotoxicity.

### 3.2 XPP inhibited peanut specific IgE production and protected PN allergic mice against PN anaphylaxis

The *in vitro* data above prompted us to test whether XPP inhibits IgE production *in vivo*. We employed a well-established PN allergy model in which B and T cell responses and clinical reactions resemble human peanut- allergy. This model has been used by us and many other investigators (29, 36). We tested XPP effect in two separate experiments in which mice were either systemically sensitized with PN+ alum adjuvant (Model # 1, **Figure 1A**) or orally sensitized with PN + cholera toxin (Model #2, **Figure 1B**). Both sensitization protocols are well established (28, 29, 36, 40). To elicit anaphylaxis, all mice were challenged orally and XPP treatment was given i.g. as indicated (**Figures 1A, B**). PN-specific IgE levels at challenge in XPP-treated mice were significantly and markedly reduced compared to the sham-treated PNA mice in both models (75% reduction, p <0.001 *vs.* Sham, **Figure 1C**). Impressively, all XPP-treated mice were completely protected from anaphylaxis following PN challenge, evidenced by symptom scores of 0 (**Figure 1D**, p <0.001 *vs.* Sham), normal body temperatures (**Figure 1E**, p < 0.001 *vs.* Sham), and essentially normal plasma histamine levels (**Figure 1F**, p < 0.001). Protection in model #1 was observed 7 weeks after stopping therapy with significantly reduced symptom scores and PN-specific IgE levels and increased body temperatures, which all indicated a persistent effect (**Supplementary Figure 3**). Results in model #2 represent efficacy of late treatment with XPP as it was administered post-boosting, at a time when mice had established food allergy and were capable of anaphylaxis (34). Finally, XPP did not reduce PN-IgG1 (**Figure 1G**), PN-IgG2a (**Figure 1H**) or PN-IgA (**Figure 1I**) in the orally sensitized mouse model #2, which demonstrated its selectivity for IgE reduction. Furthermore, correlation analysis revealed that PN specific IgE showed significant positive correlation with symptom score and plasma histamine level (r = 0.9083, p < 0.0001; r = 0.8903, p < 0.0001, **Figures 1J** and **K**) and negatively correlated with body temperatures (r = -0.8906, p < 0.0001, **Figure 1L**).

### 3.3 Oral administration of XPP has a high safety profile

Given that XPP effectively suppressed IgE production and protected peanut allergic mice from anaphylaxis, it may have a potential for clinical use. It is important to understand its safety profile. Acute toxicity and sub-chronic toxicity experiments of XPP were performed. In the acute-toxicity assay, C3H/HeJ mice were fed with 10 times (4 mg/day) of the regular XPP treatment dose once and observed daily for 14 days. No deaths and abnormal behavior or diarrhea were observed (**Table 1**). In the sub-chronic toxicity assay, mice were fed 5 times (2 mg/day) of the regular treatment dose for 14 consecutive days. No deaths and abnormal behavior or diarrhea were observed either, and all mice appeared healthy. Mice were then sacrificed, and blood samples were obtained for chemical analysis by ALX laboratories (NY). Results showed that the serum ALT and BUN levels were similar to the control group and within the normal range (**Table 1**). The complete blood count (CBC) tests showed that the white blood cell, red blood cell, hemoglobin and platelet levels in the XPP treated group were also within the normal range and similar to the control group (**Table 1**). These results demonstrate that XPP has a high safety profile. These data are in line with the *in vitro* data that demonstrated no cytotoxicity of XPP or global immune suppression.

**Table 1:** Safety evaluation of XPP.

	Naïve	XPP (Acute)	XPP (sub-chronic)	Reference
Treatment	Vehicle	10x	5x	
Mortality (%)	0.00	0.00	0.00	N/A
Morbidity (%)	0.00	0.00	0.00	N/A
BUN	27.0 ± 2.8	19.01.2	19.86±2.19	9~36
ALT	27.5 ± 10.6	24.8±11.7	23.57±9.57	22~400
RBC (M/uL)	7.17±0.43	6.93±1.11	7.34±0.40	2.8~10.8
HGB (g/dL)	11.2±0.49	10.8±1.56	11.83±0.65	3.8~10.2
PLT(K/pL)	505.00±342.52	407.25±252.86	553.67±79.34	6.3~16.3
WBC (K/uL)	6.225±2.93	4.04±0.71	2.91±0.70	115~840

For acute toxicity experiment, C3H/HeJ mice were fed 10 times normal dose (4mg/day/mouse) and observed for 14 days. For sub-chronic toxicity experiment, C3H/HeJ mice were fed 5 times of normal dose (2mg/day/mouse) for 14 days. Naive group of mice were fed with water as controls. Mice were sacrificed and blood samples were collected after each experiment. Blood urea nitrogen (BUN) and alanine aminotransferase (ALT) measurements for evaluation of kidney and liver functions respectively and complete blood count (CBC) testing were performed by ALX laboratories, NY. BUN, Blood Urea Nitrogen; ALT, Alanine Aminotransferase; RBC, Red Blood Cells; HGB, Hemoglobin; PLT, Platelets; WBC, White Blood Cells.

### 3.4 XPP decreased the IgE-expressing memory B cells in spleen and bone marrow

**Figure 3:**
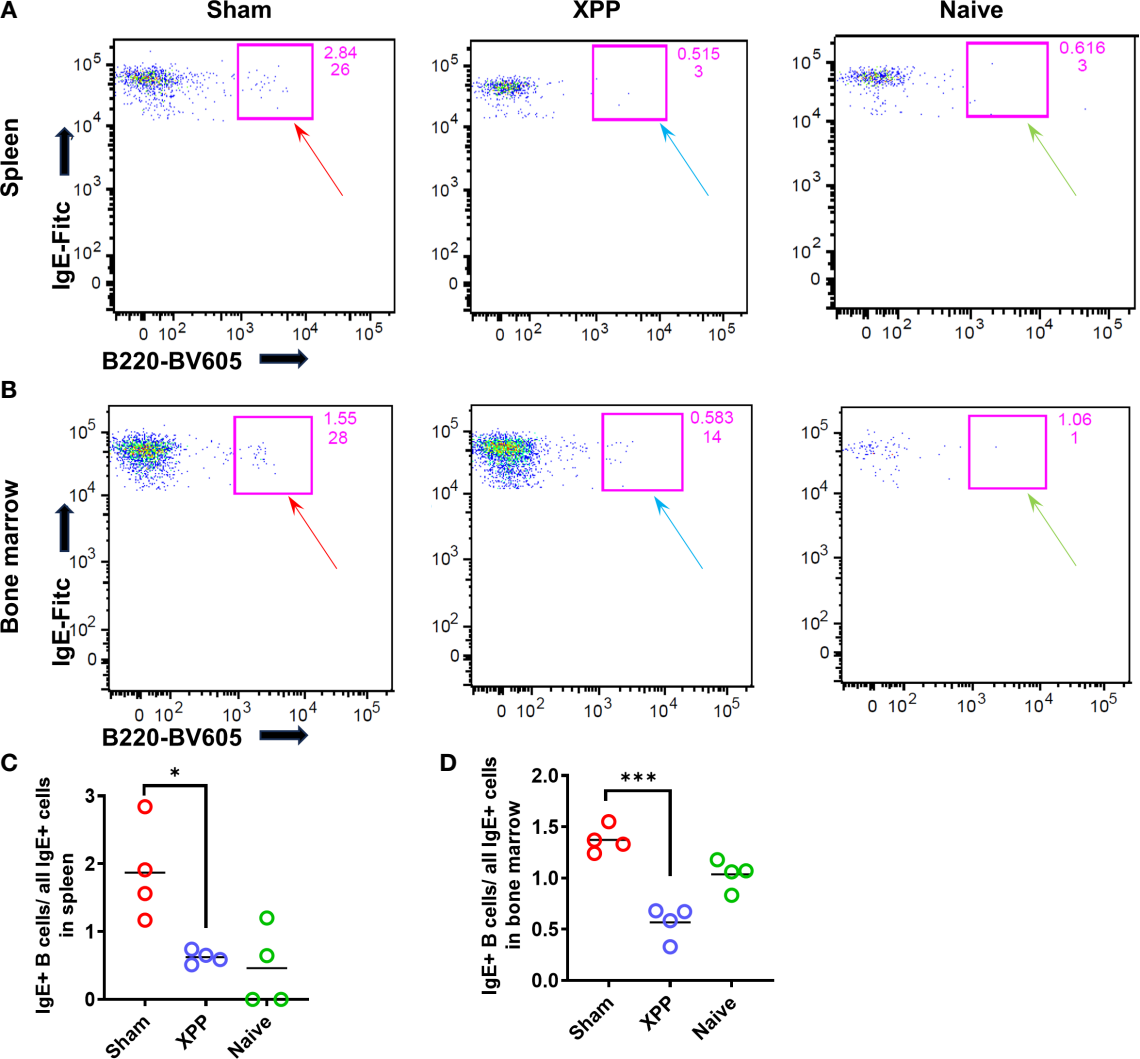
IgE+ B cells population in spleen and bone marrow samples. Splenocytes and bone marrow cells from oral sensitized model #2 were stained with antibodies to CD45R/B220 and IgE and analyzed by flow cytometry. **(A)** IgE+ B cells were found in the spleen of Sham, XPP treated, and naive mice; **(B)**; IgE+ B cells were found in the bone marrow of Sham, XPP treated, and naive mice; **(C)** Percent of IgE+ B cells in spleen; **(D)** Percent of IgE+ B cells in bone marrow; *p ≤ 0.05; ***p < 0.001, n=4.

Since XPP significantly reduced the PN-specific IgE levels compared to sham-treated mice, we, therefore, addressed questions of whether the IgE-producing B cells numbers are modified by XPP treatment. IgE-producing B cells from the spleen and bone marrow (samples collected from orally sensitized mouse model #2) were evaluated using flow cytometry of cells stained for intracellular IgE (**Figures 3A, B**). The percentage of the IgE+B cells (over all IgE+ cells) was significantly reduced by XPP in both spleen and bone marrow cells (p < 0.01; p < 0.001, **Figures 3C, D**).

### 3.5 XPP treatment reduced production of IL-4 but not IFN-γ or IL-10

**Figure 4:**
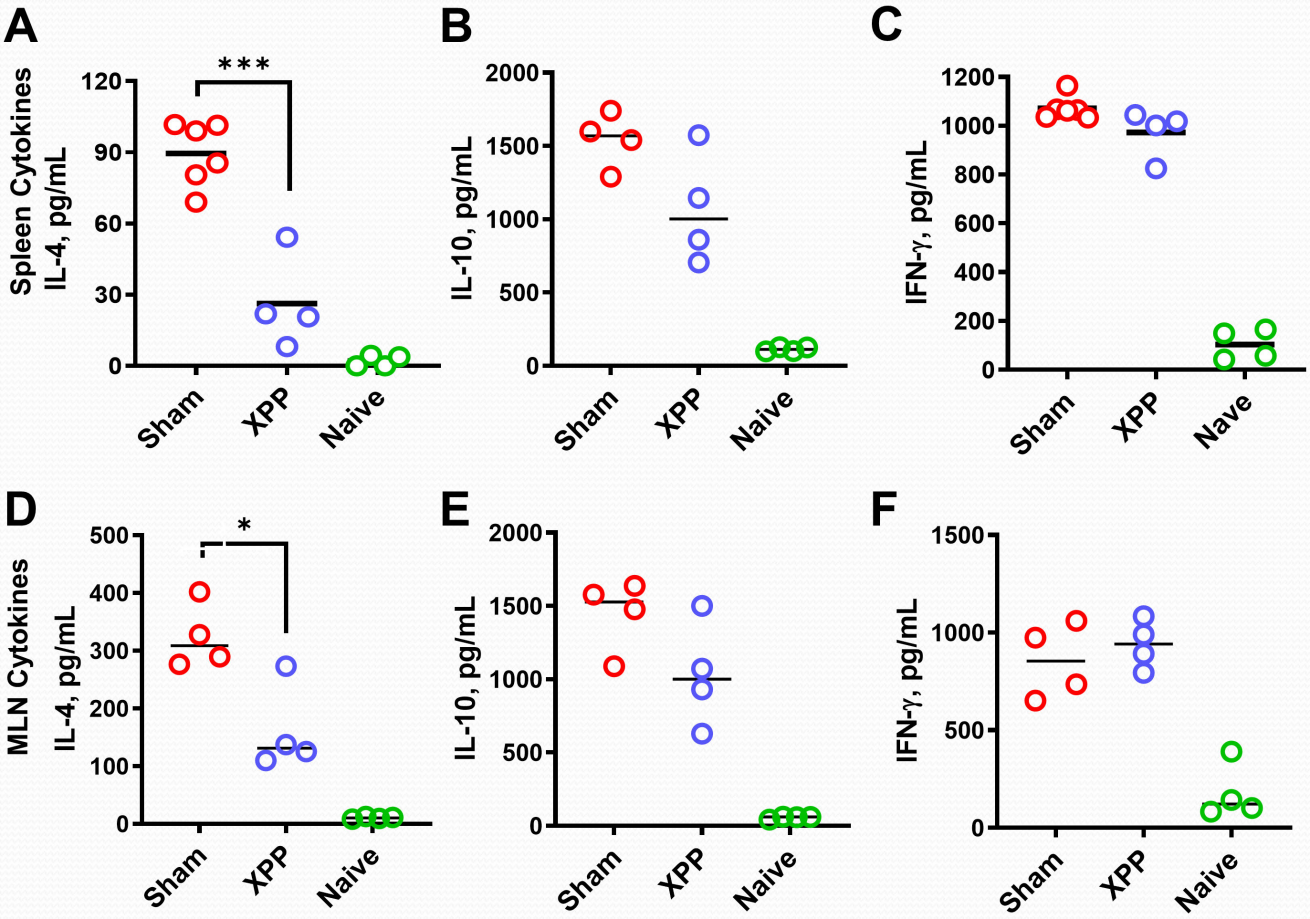
Effect of XPP on cytokine levels and IgE-producing B cell numbers. Splenocytes culture and the MLN cell cultures (from oral sensitized model #2) were stimulated with protein extracts of peanut (CPE). IL-4 **(A)**, IL-10 **(B)** and IFN-γ **(C)** production in murine splenocytes; IL-4 **(D)**, IL-10 **(E)** and IFN-γ **(F)** production in murine MLN were measured by ELISA. Bars indicate group means. N = 4-5 mice/group. *: p ≤0.05, ***: P<0.001 *vs*. Sham.

IL-4 is the key Th2 cytokine promoting IgE antibody switching and memory, while IFN- γ and IL-10 inhibit IL-4 production (41). We determined IL-4 production by splenocytes (SPCs) and MLN cells harvested from each group of mice from the orally sensitized mouse model #2 following the last challenge. XPP-treated mice showed significantly inhibited IL-4 level in both splenocyte and MLN cultures after the crude peanut extract (CPE) stimulation (**Figures 4A, D**). There were no significant differences in IL-10 (**Figures 4B, E**) or IFN**-γ** production (**Figures 4C, F**) in XPP-treated group in both the SPC or MLN cultures compared with the sham group. These data suggest that XPP may have a direct effect on IL-4 regulation.

### 3.6 XPP increased the methylation at murine IL-4 promoter

**Figure 5:**
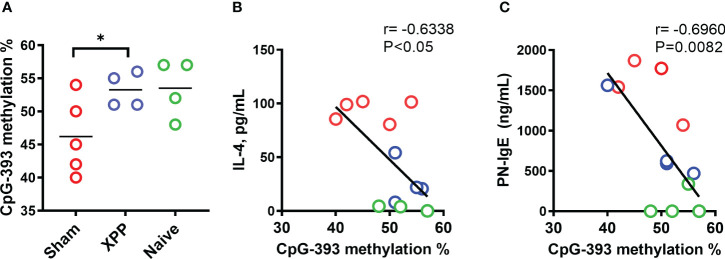
Percentage methylation of CpG sites in IL-4 promoter. Percentages of DNA methylation at the CpG-393 **(A)** site of IL-4 gene promoters in splenocytes collected from oral sensitized model #2 was assessed by pyrosequencing of bisulfite converted genomic DNA. Bars in A is group means. Pearson correlation between splenocyte IL-4 production vs. methylation percentage of CpG-393 **(B)** and PN-specific serum IgE vs. methylation percentage of CpG-393 **(C)** were calculated. N = 4-5 mice/group. * P<0.05.

Methylation status at the IL-4 promoter directly regulates IL-4 transcription; i.e. increased methylation suppresses IL-4 transcription whereas decreased methylation increases IL-4 transcription (42, 43). We therefore determined methylation levels at CpG-393 of IL-4 promoter as previously described (34). Percentages of CpG methylation for CpG-393 showed an increase in XPP-treated mice compared to sham (**Figure 5A**) suggesting that XPP treatment leads to a more closed IL-4 promoter. In all groups, methylation of IL-4 promoter CpG site showed robust significant inverse correlation with IL-4 production (**Figure 5B**, r = -0.6338, p < 0.05) and PN-specific IgE (**Figure 5C**, r = -0.6960, p < 0.0082). These data suggest that regulation of IL-4 promoter CpG methylation status may be a contributing mechanism of XPP suppression of IL-4 production.

### 3.7 Transcriptional profiling of IgE producing plasma cells treated with XPP

**Figure 6:**
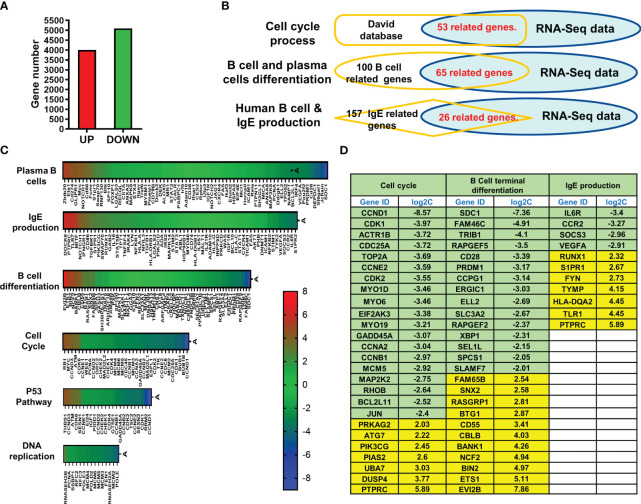
RNA sequencing of XPP treated U266 cells and pathway analysis. **(A)** Bar graph of total up-regulated a down-regulated genes; **(B)** RNA-Seq expression profile of most up- or down-regulated genes in cell cycle, B cell differentiation, and IgE production. **(C)** Heatmap of RNA-Seq results resulted to 6 pathways; **(D)** Table of candidate genes which have most significant changes. The threshold of log2C values was set as lower than -2.0 or higher than 2.0.

RNA-Seq was used to evaluate the full gene-expression profiles of XPP-treated IgE producing human plasma cells. Overall, 4009 genes were upregulated, and 5094 genes were downregulated (**Figure 6A**). To further analyze the RNA-Seq results, pathway enrichment was investigated using DAVID. 53 related genes were matched to cell cycle process using David database. 65 related genes were matched to B cell differentiation- related genes, and 26 related genes were matched to IgE-related genes from Pubmed database (**Figure 6B**). The overlap graph showed the number of genes found in our RNA-Seq data using each method. Six pathways were involved in the regulation of XPP-treated IgE-producing plasma cells. These pathways are related to plasma B cells, IgE production, B cell differentiation, cell cycle, P53 pathway, and DNA replication. The heatmap for each pathway is shown in **Figure 6C**. As hundreds of genes were identified in the six enriched pathways, we further narrowed down candidate regulatory genes by filtering for differential expression using Log2C values > 2 or < -2 as cut-off values. Upregulated genes are shown in yellow whereas downregulated genes are shown in green (**Figure 6D**).

### 3.8 PCR validation of candidate genes related to cell cycle, B cell differentiation, and IgE production in XPP treated IgE-plasma cells

**Figure 7:**
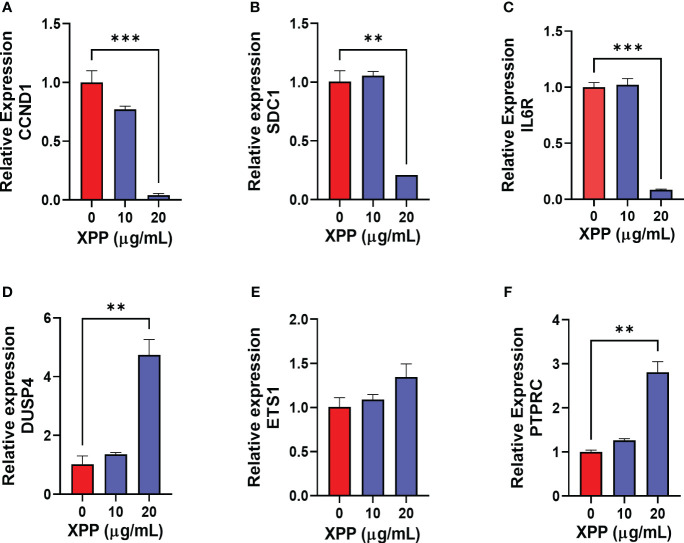
Relative gene expression of 6 down-or up-regulated genes in XPP treated U266 cells. The relative expression level of CCND1 **(A)**, SDC1 **(B)**, IL6R **(C)**, DUSP4 **(D)**, ETS1 **(E)**, and PTPRC **(F)** were determined by comparing with GAPDH mRNA expression. **p<0.01 ***p<0.001.

In order to further verify XPP mechanism of action at the molecular level, the expression levels of some of these target genes were validated by qRT-PCR. We chose the CCND1, DUSP4, and PTPARC from the cell cycle related genes, SCD1 and ETS1 from the B cell terminal differentiation gene list, and IL6R and PTPRC from the IgE production related gene list as the targets for RT-PCR validation. The RT-PCR confirmed that the CCND1, SCD1, and IL6R genes expression were significantly reduced in U266 cells with the treatment of XPP at the concentration of 20 μg/mL (**Figures 7A–C**, p < 0.001), while the DUSP4 and PTPRC gene expression was significantly increased by XPP at the same concentration (**Figures 7D, F**, p < 0.01). ETS1 gene expression also showed the upregulation trend with the increasing concentration of XPP, but no significant value was observed (**Figure 7E**). Overall, we were able to identify key genes associated with different pathways involving disease progression, and successfully validated some of these genes by qRT-PCR.

## 4 Discussion

Efficacious reduction of IgE for the treatment of food allergy remains elusive. Herbal medicines have been investigated for their inhibition effect on IgE production. We previously showed that the herbal formula FAHF-2 and its refined form showed significant IgE inhibition, both *in vitro* and *in vivo* (44–46). The active compound, berberine, was isolated from *P. chinensis*, which is one of the nine herbal constituents in FAHF-2 formula (31). We showed that berberine significantly inhibited the IgE production *in vitro* and modulated the ε-germline transcript expression through regulation of the phosphorylation of IƙBα, STAT-3 and T-bet (31). However, the bioavailability of berberine is poor. Oral berberine alone did not reduce PN-specific IgE levels and failed to protect PN-allergic mice from anaphylaxis, while the whole formula showed significant suppression of IgE levels (47).

Previously, our lab determined that the herbal medicine, *R. cordifolia*, inhibited the IgE production both *in vitro* and *in vivo* (28). *R. cordifolia* also reduced the anaphylactic symptoms in PN allergic mouse model (28). Historically, *R. cordifolia* is a widely used herbal medicine in Asia. Recent studies have shown that *R. cordifolia* roots have antibacterial, antioxidant and anti-inflammatory activities (28). *R. cordifolia* is enriched for anthraquinones, which have been isolated and identified, such as, alizarin (1,3-dihydroxy-2-ethoxymethyl-9,10 anthraquinone), purpurin (1,2,4-trihydroxyanthraquinone), mollugin (1-hydroxy-2-methy-9,10-anthraquinone) (48–50). We, for the first time, investigated the effect of xanthopurpurin on PN specific food allergy using murine models. Importantly, complete protection from anaphylaxis persisted for 7 weeks after stopping treatment which is longer than observed for OIT approaches in murine models (**Supplementary Figure 3**) (34). Protection was also observed when late treatment XPP was given after establishing PN allergy. Other compounds from *R. cordifolia*, such as purpurin and alizarin, inhibited IgE production only *in vitro*, but failed *in vivo* (Data not shown). Xanthopurpurin (XPP) significantly inhibited IgE production not only *in vitro*, but also *in vivo*, similar to the whole *R. cordifolia* herbal medicine (28). Thus, XPP has promise as a therapy for food allergy.

Food-induced anaphylaxis is an IgE-dependent type-I hypersensitivity reaction. Allergen-specific IgE produced by B cells is increased in response to allergen stimulation due to increased number of IgE-producing B cells. Regulation of IgE levels using anti-IgE molecules has been employed in many studies targeting various allergic diseases (26, 51). However, omalizumab can neutralize the free serum IgE (52), but less information about targeting to IgE production B cells and plasma cells. Compared with anti-IgE therapies, the XPP treatment not only inhibited IgE production, but it also suppressed the number of IgE producing B cells in our animal model. Downregulation of IgE-producing B cells provides evidence of a direct effect on IgE production.

Food allergy is associated with the imbalance of Th1and Th2 response. Th2 response was dramatically increased while Th1 response was diminished in food allergy (53). Th2 cytokines play important role in the pathogenesis of food allergy. IL-4 is pivotal for the activation of Th2 response and INF-γ is associated with the active Th1 response. To investigate the effect of XPP on PN allergic, we utilized two mouse models, systemic model and oral model, in this study. Both systemic model (i.p. sensitization) and oral model (i.g. sensitization) induced persistent peanut hypersensitivity. Systemic model using i.p. sensitization method in C3H/HeJ mice is most efficient, fast and simple to achieve, with high levels of PN sIgE and hypersensitivity. Oral model with i.g sensitization is more similar to the real food allergy scenario on human who was exposed to the PN allergen by food consumption. The oral model required to use the cholera toxin as the adjuvant, which is toxic, and need a longer time to introduce the allergic reactions. In this study, we first tested XPP on a fast established systemic sensitization model. Then, we investigated the effect of XPP on oral sensitization model which mimic the peanut allergy in reality. XPP treatment reduced IgE production, but did not show obvious effects on PN-sIgG1, -sIgG2a, and -sIgA levels (**Figures 1G–I**). IgG2a is Th1-associated immunoglobulin. Our data showed that the XPP treatment significantly suppressed the IL-4 production, but did not significantly inhibit the production of IL-10 and INF-γ. We also performed the *in vitro* experiment of XPP on human IgG producing myeloma cell line, ARH-77 (ATCC, Manassas, VA). XPP didn’t show significant inhibition on the IgG production. No toxicity was observed in this experiment (**Supplementary Figure 4**). All these data together suggest that XPP treatment selectively regulates Th2 response, without global immunosuppression. However, whether XPP has an effect on other IgG-producing myeloma cells lines, or on IgA- IgM- or IgD-isotype antibody producing myeloma cells are unknown, requiring further investigation.

Epigenetic mechanisms have been suggested in food allergy studies (54). Prior studies reported that allergic subjects showed significantly lower methylation level at IL-4 promoter region compared with health subjects (55). We found that XPP treatment increased DNA methylation status of CpG at site -393 compared with the sham group (p = 0.05). Decreased DNA methylation in Sham group is consistent with higher IL-4 gene expression, whereas higher DNA methylation in XPP-treated group implies decreased transcription of IL-4, which in turn reduces production of IL-4. Correlation data also confirmed that the increased DNA methylation at CpG-393 was negatively correlated with IL-4 expression and IgE production.

Additional mechanisms contributing to the regulatory effect of XPP on IgE production may involve targeting of genes related to other signal pathways. Our RNA-seq data of XPP-treatment on U266 cell identified 6 pathways/processes as being enriched, including Plasma/B cells and IgE production, among others. Significantly up or down regulated genes (log 2C value > 2 or < 2) were selected as candidates for the validation using qPCR. Top 3 down regulated and 3 upregulated genes showed CCND1, SDC1 and IL-6R were significantly reduced whereas DUSP4 and PTPRC expressions were significantly increased. The ETS1 gene showed mild non-significant increase at 20 μg/mL of XPP. Previous reports of these genes in the context of food allergy are scarce. CCND1 and IL-6R have been reported to be associated with asthma inflammation and high serum IgE in patients (56–58). IL-6 signaling directly related to the survival and maturation of B cells and Plasma cells (59, 60). SDC1 is a maker of long-lived plasma cells and myeloma pathogenesis (61, 62). DUSP4, on the other hand, was reported as a tumor suppressor (63, 64). DUSP4 expression reduced the expansion of antigen-specific B cells and the production of antibodies (65). While the direct immunoregulatory targets of XPP remain to be identified, our data suggests that the XPP treatment decreases Th2-immune responses and pathways related to IgE production.

In conclusion, we, for the first time, demonstrate that xanthopurpurin, an active compound isolated from *R. cordifolia*, suppressed the IgE production *in vivo* and protected peanut allergic mice from anaphylactic reactions. Furthermore, xanthopurpurin also exhibits regulatory effects on IL-4 promoter DNA methylation, and on expression of several genes related to B cell survival and IgE production.

## Data availability statement

The original contributions presented in the study are included in the article/**Supplementary Material**. Further inquiries can be directed to the corresponding authors.

## Ethics statement

Ethical approval was not required for the studies on humans in accordance with the local legislation and institutional requirements because only commercially available established cell lines were used. The animal study was approved by Institutional animal care and use committee (IACUC) at Icahn Mount Sinai School of Medicine, New York. The study was conducted in accordance with the local legislation and institutional requirements.

## Author contributions

NY: Conceptualization, Data curation, Formal analysis, Investigation, Methodology, Project administration, Supervision, Writing – original draft, Writing – review & editing. KS: Conceptualization, Data curation, Formal analysis, Investigation, Methodology, Project administration, Validation, Writing – review & editing. YC: Data curation, Formal analysis, Investigation, Methodology, Writing – review & editing. HL: Data curation, Investigation, Methodology, Writing – review & editing. AM: Data curation, Investigation, Methodology, Writing – review & editing. PY: Data curation, Investigation, Methodology, Writing – review & editing. XL: Conceptualization, Writing – review & editing. RT: Writing – review & editing. JG: Writing – review & editing. AN: Writing – review & editing. JZ: Data curation, Writing – review & editing. X-ML: Conceptualization, Data curation, Formal analysis, Funding acquisition, Investigation, Methodology, Project administration, Resources, Supervision, Writing – review & editing.
