# Supplementary material for: Sustained silencing peanut allergy by xanthopurpurin is associated with suppression of peripheral and bone marrow IgE-producing B cell
Source: Front Immunol. 2024 Feb 6;15:1299484. doi: 10.3389/fimmu.2024.1299484 (PMC10876879; doi:10.3389/fimmu.2024.1299484)
Supplement: Supplementary file 1 [file DataSheet_1.docx]

**Supplementary data**

**Method 1: IgG production by ARH-77**

ARH-77, a human IgG-producing myeloma cell line, was purchased from ATCC (American Type Culture Collection; Manassas, VA). The culture media contains RPMI 1640 medium supplemented, 10% FBS, 1 mM sodium pyruvate, 1×10^-5^ M β-ME and 0.5% penicillin-streptomycin. Cells were cultured at 37°C under 5% CO2. 2×10^5^ cells/mL were co-incubated with different fractions of *R. cordifolia* at 10 μg/mL on day 0. After 3 days, supernatants were harvested and IgG levels were determined using an ELISA Kit (Mabtech Inc, OH).

**Method 2: Cell viability:**

Cell viability was evaluated using the trypan blue exclusion assay. Briefly, 10µL of ARH-77 cells at each testing condition was mixed with 10 µL of trypan blue dye. The mixture was then loaded into a hemocytometer and the viable cells were counted under a microscope. The percentage of viable cells was calculated as follows: Viable cells (%) = (total number of viable cells) / (total number of cells) × 100.

**Supplementary data figure legends:**

**S Figure 1. Singlet cells were selected on the basis of FSC-A/FSC-H profile.** From singlet cells, lymphocytes were gated based on forward and side scatter properties. From this gate, live cells were selected as cells negative for Live-Dead stain. From live cell gate, all IgE+ (Fitc-IgE) cells were gated and subsequently analyzed for IgE+ B cells (Fitc-IgE +; BV605-B220+ cells).
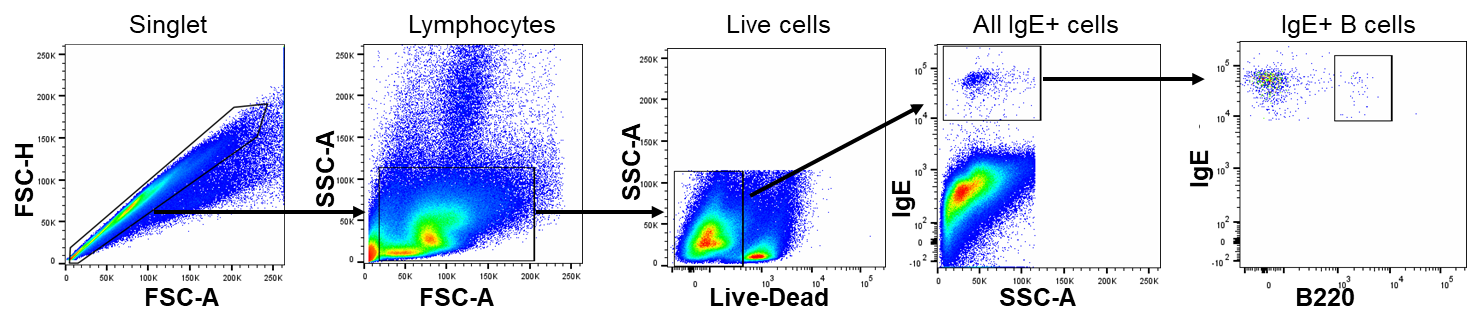


**S Figure 2. The IgE inhibitory effect of five fractions from *R.cordifolia*.** U266 cells (2 × 10^5^ cells/mL) were co-incubated with each fraction at the concentration of 10 μg/mL for 6 days. The supernatants were harvested and IgE levels were measured by ELISA. The inhibition percentage was calculated using the equation “% inhibition = (1- IgE level _each fr_ / IgE level _non-treated_ ) x 100%”.

**
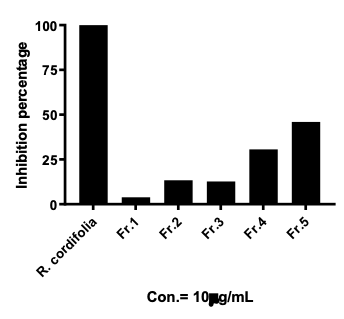
**

**S Figure 3. Sustained suppression of XPP against PNA anaphylaxis.** In model #1, 7 weeks after the therapy, mice were orally challenged with PN extract. The symptom scores, PN specific IgE levels and body temperatures were measured. A. Symptoms scores, B. PN specific IgE. C. Body temperature. ***=P<0.001 vs. sham. N=4 or 5 mice/group.


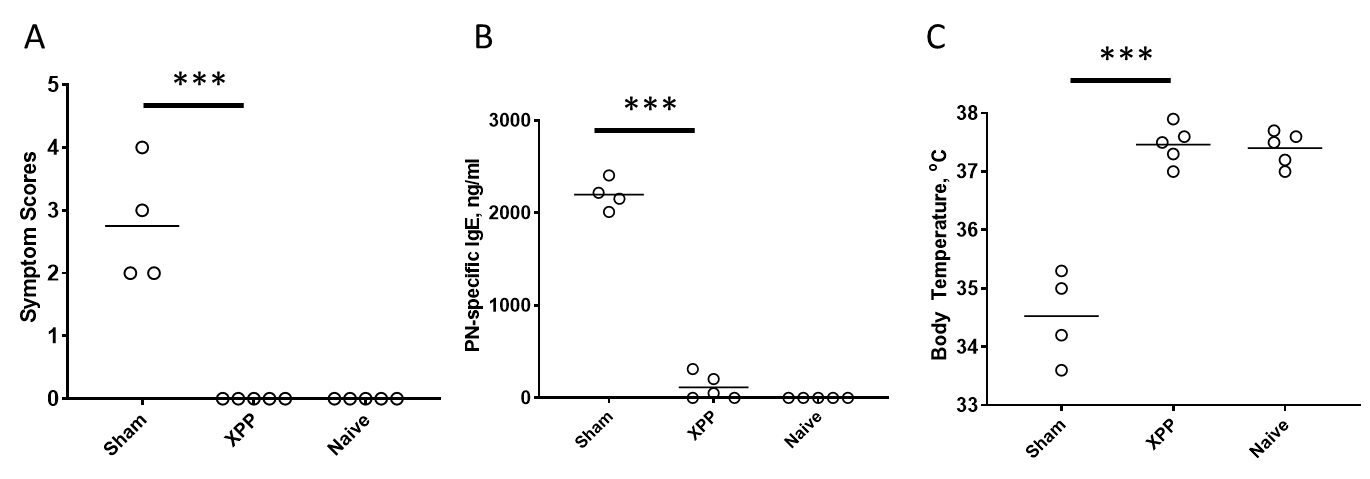


**S Figure 4. The effect of XPP on IgG production by ARH-77 cells.** A. IgG level in ARH-77 cells treated with XPP at the concentrations of 2.5, 5, 10, 20, and 40 μg/mL. B. Cell viability of ARH-77 cells treated with different concentrations of XPP.

**
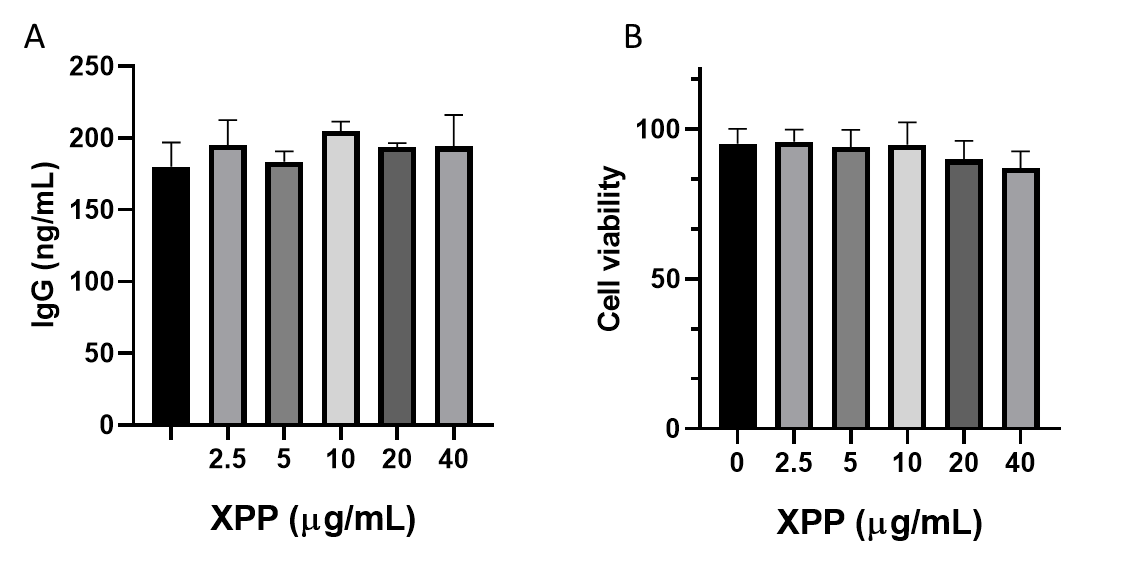
**

**S Table 1: Primer sequences of CCND1, SDC1, IL6R, DUSP4, ETS1, PTPRC, and GAPDH**.


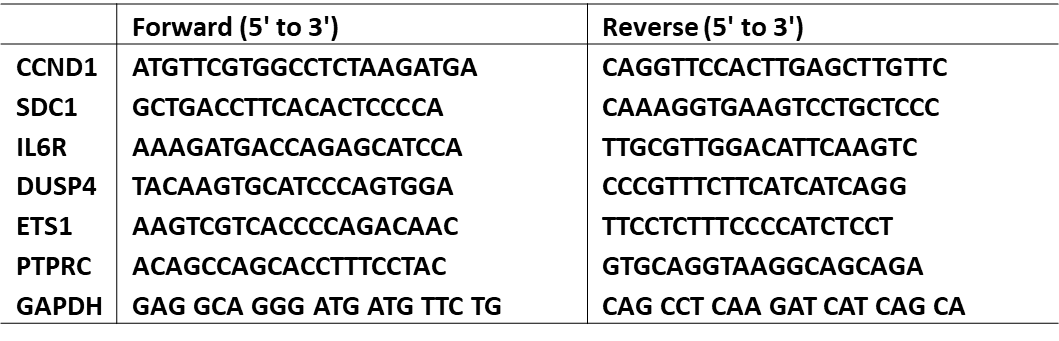


**S Table 2: 1H NMR and 13C NMR data of XPP**

**
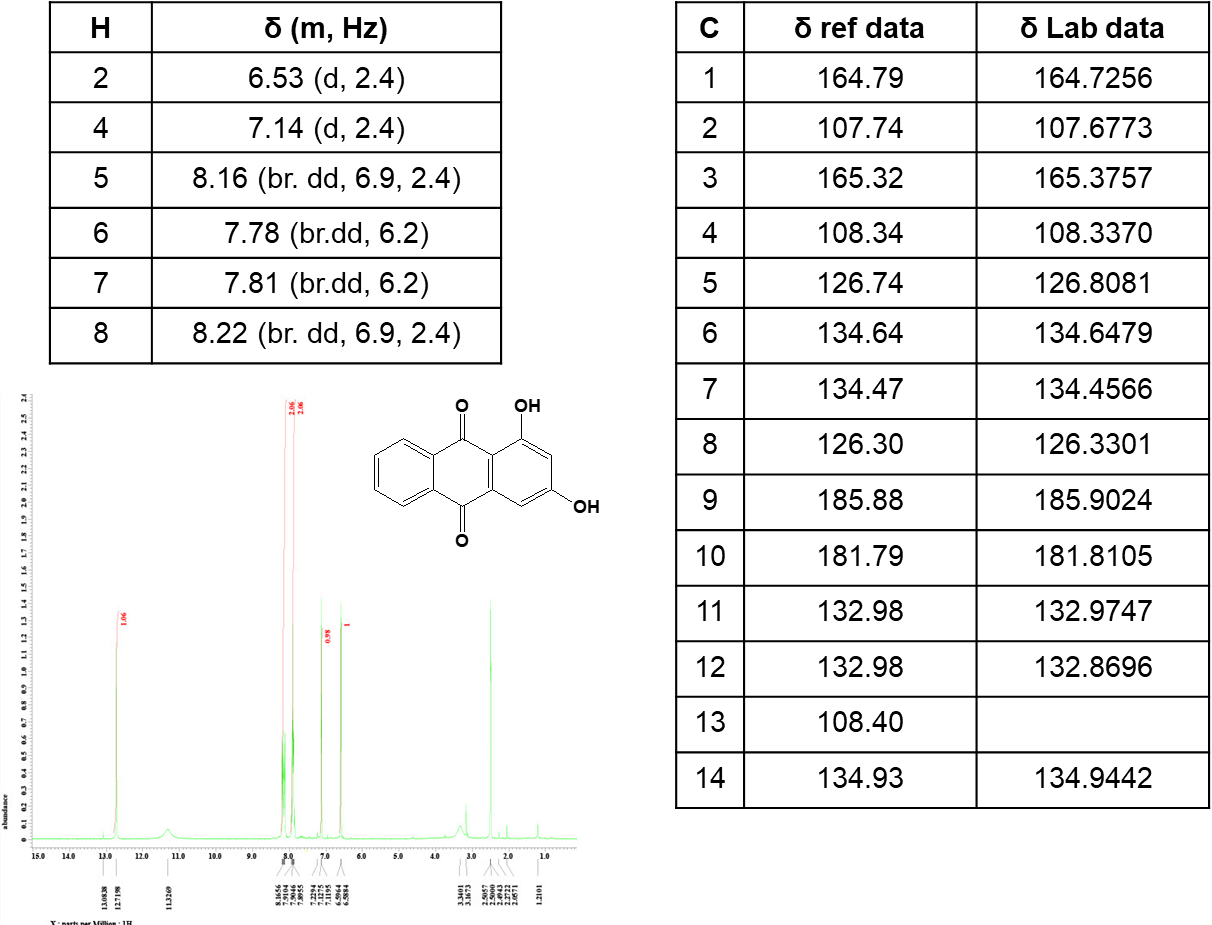
**
